# Single-Site Experience with an Automated Artificial Intelligence Application for Left Ventricular Ejection Fraction Measurement in Echocardiography

**DOI:** 10.3390/diagnostics13071298

**Published:** 2023-03-30

**Authors:** Krunoslav Michael Sveric, Roxana Botan, Zouhir Dindane, Anna Winkler, Thomas Nowack, Christoph Heitmann, Leonhard Schleußner, Axel Linke

**Affiliations:** Department of Internal Medicine and Cardiology, Herzzentrum Dresden, Technische Universität Dresden, Fetscherstr. 76, 01307 Dresden, Germany

**Keywords:** seamless, artificial intelligence, left ventricular ejection fraction, echocardiography

## Abstract

Left ventricular ejection fraction (LVEF) is a key parameter in evaluating left ventricular (LV) function using echocardiography (Echo), but its manual measurement by the modified biplane Simpson (MBS) method is time consuming and operator dependent. We investigated the feasibility of a server-based, commercially available and ready-to use-artificial intelligence (AI) application based on convolutional neural network methods that integrate fully automatic view selection and measurement of LVEF from an entire Echo exam into a single workflow. We prospectively enrolled 1083 consecutive patients who had been referred to Echo for diagnostic or therapeutic purposes. LVEF was measured independently using MBS and AI. Test–retest variability was assessed in 40 patients. The reliability, repeatability, and time efficiency of LVEF measurements were compared between the two methods. Overall, 889 Echos were analyzed by cardiologists with the MBS method and by the AI. Over the study period of 10 weeks, the feasibility of both automatic view classification and seamlessly measured LVEF rose to 81% without user involvement. LVEF, LV end-diastolic and end-systolic volumes correlated strongly between MBS and AI (R = 0.87, 0.89 and 0.93, *p* < 0.001 for all) with a mean bias of +4.5% EF, −12 mL and −11 mL, respectively, due to impaired image quality and the extent of LV function. Repeatability and reliability of LVEF measurement (*n* = 40, test–retest) by AI was excellent compared to MBS (coefficient of variation: 3.2% vs. 5.9%), although the median analysis time of the AI was longer than that of the operator-dependent MBS method (258 s vs. 171 s). This AI has succeeded in identifying apical LV views and measuring EF in one workflow with comparable results to the MBS method and shows excellent reproducibility. It offers realistic perspectives for fully automated AI-based measurement of LVEF in routine clinical settings.

## 1. Introduction

Echocardiography (Echo) plays a key role in assessing the left ventricular ejection fraction (LVEF) in daily practice. The measurement of the LVEF based on the modified biplane Simpson (MBS) technique is recommended by the joint American and European guidelines for LVEF calculation in two-dimensional Echo [1]. However, it is operator dependent and has a non-negligible variability, which may influence the clinician’s therapeutic decisions [2,3,4,5]. Recent technological developments in artificial intelligence (AI) have enabled automated workflows for view selection and LV function assessment [6,7,8]. Still, it is a technique that has not yet found widespread application in the daily clinical practice of Echo. This circumstance is likely due to skepticism, as only very few of these AI-based applications have been tested in a real-life clinical setting [6]. In addition, unresolved problems, such as the dependence on ultrasound systems or the inability to process analysis results offline on a workstation, may limit its attractiveness for use in clinical routines [6,7]. Recently, a commercially available and ready-to-use AI-based application for fully automated LV classification and quantification without human interaction (LVivo Seamless^TM^, DIA Imaging Analysis, Beer-Sheva, Israel) has been developed to address exactly these issues. Its accuracy has been validated with CMR imaging, but only on selected patients [9]. To address this issue, we sought to prospectively evaluate the applicability and feasibility of this AI-based application in a real-world clinical setting on a large, unselected cohort of patients. Furthermore, we wanted to compare the resulting biplane LVEF with the manual MBS technique and, if we found there to be a difference between them, elucidate the underlying mechanism. We also aimed to determine the reproducibility and repeatability of the AI techniques in relation to the use of the MBS method in successive Echo examinations.

## 2. Methods

### 2.1. Study Design

From the beginning of February 2022 to the end of March 2022, we consecutively enrolled adult patients (age > 18 years) who were referred to our department for clinically indicated Echo examinations as part of a diagnostic or therapeutic procedure. Patients from the intermediate and intensive care unit or the emergency ward were excluded. All examinations were performed by one of the three experienced cardiologists in the Echo laboratory of the Department of Internal Medicine and Cardiology, Dresden Heart Centre, Dresden University of Technology, Germany.

### 2.2. Echocardiographic Imaging and Analysis by Cardiologists Using MBS Method

Patients received a standard Echo examination performed using EPIQ CV devices (Philips Healthcare, Andover, MA, USA) by the designated cardiologists according to the current guidelines [1]. The examiner acquired the intended clips, stored them locally on the Echo machine, and eventually performed the required measurements and, using the MBS technique, determined biplane LVEF to be the difference between end-diastolic volume (EDV) and end-systolic volume (ESV) relative to the EDV [1]. Measurements were averaged over two cardiac cycles for patients in a sinus rhythm; otherwise, three cardiac cycles were analyzed according to standard practice at the given institution. If one of the two apical views was not available or not analyzable with the MBS method despite the use of ultrasound enhancing agents (*n* = 65), the examination was excluded from further analysis. The assigned cardiologists had knowledge of the patients’ clinical status but were initially blinded to the results of the subsequent AI analysis. All results from the online analysis were stored as structured reports in a separate database and the clips were sent to the virtual server hosting the AI (see below). The acquired data were stored automatically, excluding the possibility of adjusting measurements after the completion of the Echo study.

### 2.3. Briefing and Self-Study of Participating Cardiologist

The participating cardiologists received a briefing session shortly before and four weeks after study enrollment began. The main focus was on acquiring Echo clips that include the entire left ventricle in the apical 4- and 2-chamber views, on adjusting depth so that two-thirds of each view would be occupied by the left ventricle, and on aligning the interventricular septum parallel to the image plane. Imaging settings needed to be optimized for endocardial visualization. At the end of the shift, each of the three participating cardiologists had to review and evaluate for self-study the previous results of AI classification and tracking, as well as the previous Echo examinations he/she had performed. It was not possible to change or adjust the previously measured results.

### 2.4. Analysis by Artificial Intelligence Application

After the MBS measurements were determined online using the ultrasound machine, the complete Echo exam with all clips was sent in a Digital Imaging and Communications in Medicine (DICOM) format to our in-house medical server virtually hosting LVivo Seamless^TM^ (DIA Imaging Analysis, Beer-Sheva, Israel) via the standard Picture Archiving and Communication System (PACS). The LVivo Seamless^TM^ system is a vendor-neutral, commercially available, Food and Drug Administration (FDA)-cleared, and conformité européenne (CE)-marked AI-based application. According to the manufacturer, this AI-based application has been trained with over 100,000 frames from different patients with normal and abnormal LV function and various pathologies, and from different ultrasound devices. The AI scans the entire Echo exam and automatically selects the optimal LV 4- and 2-chamber views using an algorithm that is based on a convolutional neural network (CNN). Since this particular AI-based application is patented, we have no further knowledge about the underlying architecture of the CNN. However, a recent review provides a brief introduction to the field of machine-/deep-learning-based applications in medical imaging [10]. After the clips are selected, the LVivo Seamless^TM^ automatically activates the LVivo EF^TM^ module to calculate EDV, ESV, and LVEF through the detection and tracking of the LV endocardial border of all heartbeats of the clip. The results are sent back to the PACS server and are made available on the workstation within 4–6 min as part of the study review. The AI system provides tools on the workstation for manually editing the automatically generated results and for manually initiating (i.e., annotation) an analysis of apical LV views that were discarded by the AI in the previous seamless procedure. Although this AI application has an option for manually editing or tracing the endocardium, it was not applied in this study. Only biplane results from fully automated tracking (auto) and manual initiation were used for further analyses in this study. If the clips were still not identified, the entire exam was excluded from further analysis. An independent cardiologist who had been blinded to the MBS measurements supervised the results from the AI and performed the manual initiation of LV clips.

### 2.5. Categorization for Subgroup Analyses

To assess if the inter-method agreement was affected by the main clinical diagnosis, subgroup analyses were performed by categorizing levels of LVEF, presence of sinus rhythm, and sex. LVEF was categorized based on the standard MBS method as follows: normal > 49%, mildly reduced 49% to 40%, moderately reduced 39% to 30%, and severely reduced < 30%. Furthermore, we assessed procedural features such as image quality, operator dependency, method of AI analysis (auto vs. manual initiation) and weekly progress of the study. The image quality of all exams was classified subjectively as: good, fair, poor, or not analyzable.

### 2.6. Repeatability and Reliability

In a randomly selected subgroup of 40 patients, a complete Echo restudy was performed within one day by an independent cardiologist without alteration of patients’ hemodynamics or therapy. Each study was subsequently analyzed by an independent operator not involved in the first Echo exam in order to best reflect daily clinical practice. This independent operator was blinded to previous measurements and results. MBS measurements were performed online, stored separately and sent to the server hosting the seamless AI application for automated analysis in the same manner as the first measurement. The AI results were stored without manually adjusting the tracings. We calculated the time required for operator-dependent biplane MBS tracking and obtained the duration from sending the Echo exams to the AI analysis and presentation of results. The duration of the automated analysis time was retrieved from our hospital’s information technology department.

### 2.7. Statistics

Results are reported as medians with interquartile ranges [IQR] or frequencies with percentages (%). Inter-technique comparisons used Pearson’s correlation coefficient (R), intraclass correlation coefficient (ICC), and Bland–Altman analyses to determine agreement and consistency, mean bias, and limits of agreement (LOA) in units [11]. Positive biases represent underestimation by the AI technique. Test–retest variability was assessed by the coefficient of variability (COV), defined as the absolute difference of the corresponding pair of repeated measurements in a percentage of their mean for each patient and then averaged over the study group. For the reproducibility analysis, we calculated a sample size of 30 with an ICC of 0.91 [9] and a null hypothesis value of ICC = 0.79 with power 0.80 and alpha 0.05 (R package “ICC. Sample. Size”). The significance of measurement comparisons was assessed by paired or unpaired Student’s *t* test or Wilcoxon’s signed-rank test as appropriate after normality testing (Kolmogorov–Smirnov test). The Kruskal–Wallis test was assessed for multiple unpaired groups. Significance criteria in multiple testing were adjusted with the Bonferroni method. Differences between categorical variables or groups were assessed using chi-square tests. Statistical significance was defined as having a two-tailed *p* value < 0.05. All analyses were conducted with R (version 3.0.2, 2013, The R Foundation for Statistical Computing, Vienna, Austria).

## 3. Results

### 3.1. Study Population

Table 1 summarizes the anthropomorphic and clinical characteristics of the patients. The median age was 71 years with a prevalence of the male sex (61%), and 75% of patients had a sinus rhythm. We encountered a wide spectrum of LVEF values, ranging from 10 to 76%, as determined by the MBS method. The most common Echo indications were patients with known coronary heart disease (27%), valvular heart disease (20%) and non-ischemic cardiomyopathy (10%).

### 3.2. Feasibility

**Figure 1:**
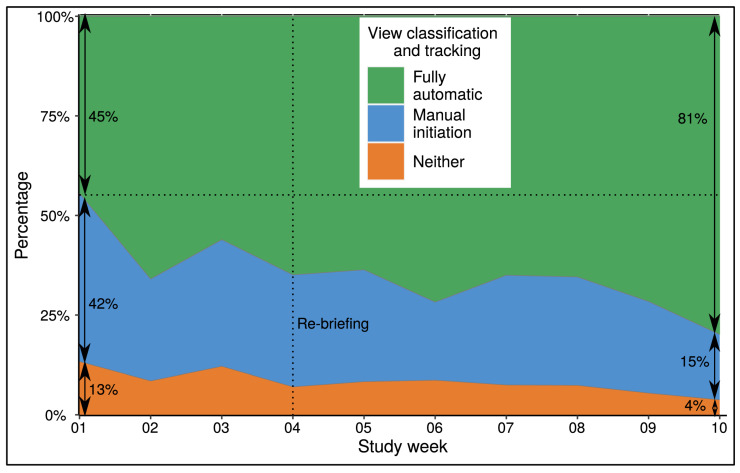
**Feasibility of Seamless and Fully Automatic View Selection of LVEF Measurement (*N* = 1083).** Improving feasibility of fully automatic view selection and LVEF measurement (green) by the AI-based application over the study period of 10 weeks. Manual initiation (blue) was performed in Echo exams with either automatically detected or measured 2- or 4- apical LV chamber views. AI = artificial intelligence; Echo = echocardiography; LVEF = left ventricular ejection fraction.

Complete standard Echo examinations were performed on 1083 patients, of which 1007 could be analyzed with the MBS method. The main reasons for excluding 76 patients from further analysis were the complete absence of an acoustic window (*n* = 22, 29%) or the severely impaired visualization of the endocardium (*n* = 54, 71%). As depicted in Figure 1, over the course of 10 weeks, the proportion of fully automated (i.e., without user involvement) selections with the calculation of biplane LVEF measurements by the AI increased, while the proportion of manually initiated or rejected Echo exams decreased substantially (*p* < 0.01 for all). In detail, the AI automatically analyzed both 4- and 2-chamber views in 675 patients (67%), only 4-chamber views in 201 patients (20%), only 2-chamber views in 60 patients (6%) and neither views in 71 patients (7%). Despite manual initiation in these 332 patients, 118 could not be analyzed by the AI and were therefore not included in the further analysis. Thus, a total of 889 Echos were successfully analyzed by both cardiologists and AI (88%). The median time for the operator-dependent MBS tracking was 171 s (IQR 147 to 206) and 258 s (IQR 240 to 276) for the fully automated AI-based method. The correct classification rate of the AI application for the LV 4- and/or 2-chamber views was 100% and we detected no erroneous tracings.

### 3.3. Measurement Comparison between Cardiologists and AI

**Figure 2:**
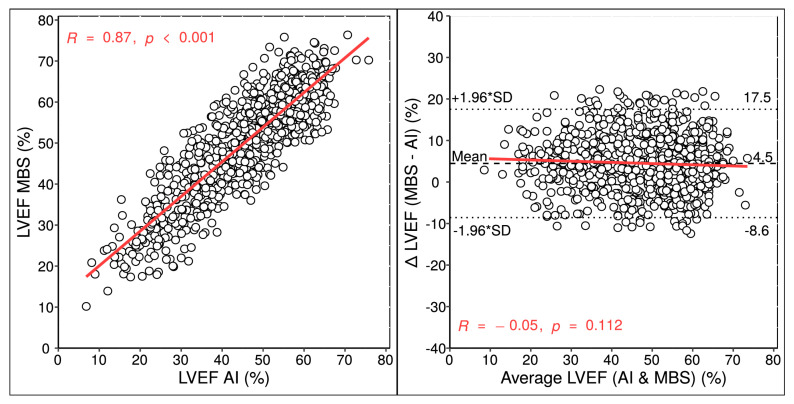
**LVEF Validation among the AI-based Application and MBS Method (*N* = 889).** Correlation (**left**) and Bland–Altman analyses (**right**) of the LVEF between the AI-based application and MBS technique with mean values (dashed horizontal lines) and upper and lower limits of agreement (dotted horizontal lines), denoted as ±1.96 * standard deviation (SD). R denotes Pearson’s correlation coefficient with corresponding significance value *p*. AI = artificial intelligence; LVEF = left ventricular ejection fraction; MBS = modified biplane Simpson.

There was a highly significant correlation of LVEF between the two methods (ICC 0.90; *p* < 0.001), and the Bland–Altman analysis of method differences revealed a mean bias of +4.5 with an estimated LOA of −8.7 and +17.6% (Figure 2). As summarized in Table 2, LV EDV and ESV correlated similarly between AI and MBS and had a similar measurement bias (−12 vs. −11 mL, *p* = 0.057), albeit with a proportional bias for increasing EDV measurements (R = 0.16, *p* < 0.001). Correlation and Bland–Altman plots for the LV volumes are provided in Figure S1.

### 3.4. Agreement and Bias Categorized Using Clinical and Echocardiographic Characteristics

**Figure 3:**
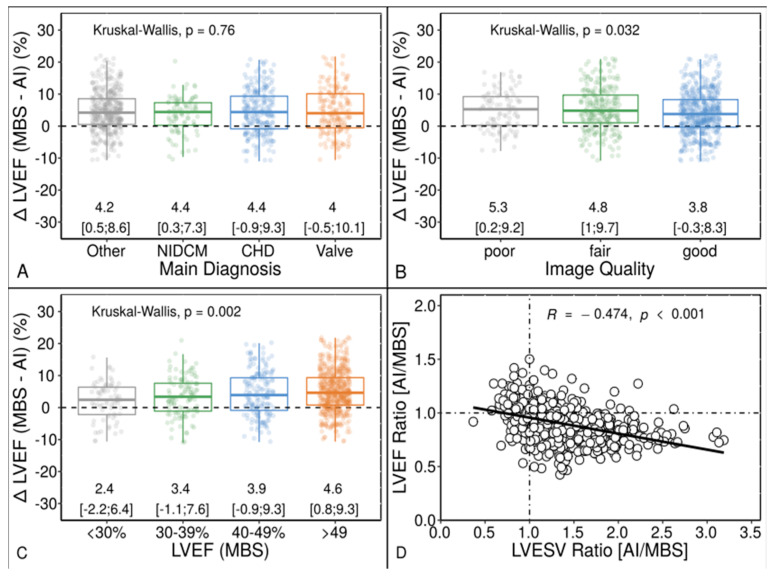
LVEF Bias Grouped by Diagnosis (**A**), Image Quality (**B**), LV Function (**C**), and by Proportion of ESV Overestimation (**D**) between the AI-based application and MBS Method (*N* = 889). Dependency of LVEF bias depicted as boxplots with jittered dots and correlation plot between the AI-based application and MBS technique. CHD = coronary heart disease; Kruskal–Wallis = Kruskal–Wallis test by ranks; NIDCM = non-ischemic dilated cardiomyopathy; Valve = severe valvular heart disease. Other abbreviations as in Figure 2.

Among patients with non-ischemic cardiomyopathy, coronary heart disease and valvular heart disease, we found no significant difference in LVEF bias between AI and MBS when compared to the overall cohort (Figure 3). The corresponding distribution of main clinical and echocardiographic characteristics is summarized in Table 3. However, the inter-method bias of LVEF decreased with improving image quality but increased with improving left ventricular function (Figure 3).

**Figure 4:**
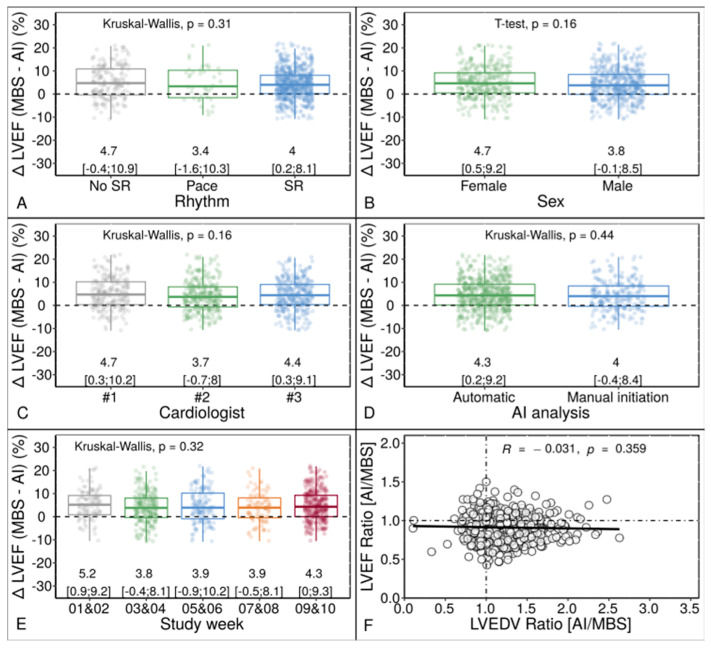
LVEF Bias Grouped by Rhythm (**A**), Sex (**B**), Operator (**C**), Type of AI Analysis (**D**), Study Period (**E**), and by Proportion of EDV Overestimation (**F**) between the AI-based Application and MBS Method (*N* = 889). Pace = ventricular pacing; SR = sinus rhythm; Kruskal–Wallis = Kruskal–Wallis test by ranks. Other abbreviations as in Figure 2.

Table 4 displays the distribution of LVEF categorization and image quality. The MBS method overestimated LVEF due to underestimating LV ESV compared to the AI-based method (R = −0.474, *p* < 0.001). The correlation plots between LV volume and LVEF differences between the methods are provided in Figure S2. Factors like atrial fibrillation, patient’s sex, and procedural aspects, such as the Echo operator, the type of AI analysis method and the timing of the Echo exam during the study, were not significant (Figure 4).

### 3.5. Comparison of Repeatability and Reliability between AI and MBS

Reproducibility and reliability results for the subgroup of randomly chosen patients for a second Echo examination are presented in Table 5. Although ICC values for both the AI-based and the MBS method ranged from excellent to good, the test–retest variability of the AI measurements was lower than of the MBS method with a COV of 3.2% vs. 5.9%, equaling a mean of 1.5% vs. 3.5% in absolute EF. Furthermore, the inter-method bias of LVEF between AI and MBS was similar to that of the main results (Figure 2).

## 4. Discussion

To our knowledge, this is the first study to demonstrate the feasibility of this novel AI-based application for fully automated view selection and classification of LVEF measurement in a clinical Echo routine. We intend to share our experiences with this particular AI and identify both its advantages and potential liabilities for use in Echo labs with a high workload. The main rationale for an AI-based workflow that operates in the background, without requiring any input from the user, is to improve accuracy, reduce measurement variability, and help researchers to conserve analysis time in the daily routine [12].

### 4.1. Feasibility

During the course of our study, we encountered many patients with a wide spectrum of cardiac diseases (Table 1 and Table 3). These patients thus exhibited a wide range of LV functions and morphology, which are necessary for clinically testing a novel AI method, especially if one intends to implement it in the Echo lab setting [13]. The overall feasibility of fully automated LV quantification by this AI is in line with the results of recent studies [6,7,8]. In addition, we detected no misclassifications of apical LV views or erroneous tracings of the endocardium, indicating a high internal quality control of the AI application. However, the underlying architecture of the CNN is not known to us as it is patented. However, this was not within the scope of our study. For a detailed background on the development of Echo and AI and currently available AI-Echo applications, including the specific application clinically tested in our study, we refer the reader to the following manuscripts [10,13]. This should not obscure the fact that a successful implementation of AI in daily Echo requires adequate use of imaging material. We learned during the study that briefing and self-study of the participating cardiologists to optimally visualize the left ventricle tremendously improved fully automated view selection and, therefore, the process of fully automated LVEF quantification (Figure 1). Even cardiologists with experience in Echo were not able to measure the biplane LVEF in ~7% of patients with the conventional MBS method due to a limited acoustic window. An alternative AI-based “eyeballing” system has been proposed to increase analysis feasibility but lacks this the ability to provide LV dimensions, representing a major drawback [14,15].

### 4.2. Comparison of LV Measurement Results

Although our experienced Echo examiners were trained and received regular briefings in order to overcome the technical obstacles in image acquisition, the measurement bias of LVEF between human operators and AI detected by our work cannot be ignored (Figure 2). Interestingly, the LVEF bias of 4.5% is congruent with the result of a previous study of this particular AI [9] and other recently published machine /deep learning-based approaches for EF quantification [4,6,7,8]. The LV volumes measured by these applications are greater than those measured by human operators using the MBS method, a finding we also observed to a similar extent in our study (Table 2). This might explain the inherent LVEF bias between AI and MBS (Figure 2). At first glance, one might assume that the LVEF level is the only explanation (Figure 3). However, we demonstrated that the LVEF difference between AI and MBS increased with deteriorating image quality, and also that LVEF bias was due to systematic underestimation of LV ESV by human operators. In other words, the smaller the ESV was determined to be by the MBS method, the greater the bias of the LVEF value compared to that of AI measurements. This suggests some subjectivity and inconsistency in endocardial tracing by human operators, as pointed out in previous studies, regardless of the imaging technique used [9,16,17].

Although the field of Echo and AI is still in its early stages, recent advances have not only focused on systolic LV function, but also on diastolic function [7], as well as taking first steps towards automated disease classification of left ventricles [8,18,19]. While our study focused on the feasibility of automated LVEF quantification, there are other potential applications of AI in Echo that can be explored. For example, AI can be used to detect specific features in Echo images, such as myocardial strain, which can provide additional diagnostic and prognostic information [20,21].

### 4.3. Repeatability, Reliability and Performance

Fully automatic AI-based applications are considered “deterministic” as they rely on mathematical algorithms to contour the endocardial boundaries, leading to identical results being obtained on the same datasets (i.e., clips) without manual contour editing, resulting in a variability of 0% [21,22]. In contrast, human operators rely on their experience and intuition, which can lead to inconsistencies in EF measurement on the same dataset [3], particularly in busy clinical settings. Variability in obtaining the 4- and 2-chamber views of the left ventricle during two-dimensional Echo, subjective assessment of the LV endocardium in systole, along with the natural variability of heart function, contributes to the measurement variance between two datasets [17,23]. Even in three-dimensional Echo and cardiac magnetic resonance imaging, which are considered the gold standards for LV function assessment, there is a test–retest variability on timely separately acquired datasets [5,24]. In the case of the AI-based method tested in our study, it is important to note that it is based on two-dimensional Echo, which has its limitations and biplane LVEF calculation relies on geometric assumptions. However, the AI-based method tested in our study provides a more consistent measurement of LVEF, with a COV of 3.2% compared to the MBS method, which has a COV of 5.9% (Table 5), which is in line with the previous reports [5,24]. Therefore, the use of AI-based methods in a clinical setting could help to reduce the variability of LVEF measurements, which may be especially beneficial for patients who require long-term LV assessment for treatment decisions.

Lastly, one could question why we did not primarily use identical echocardiographic views for the measurement of LV function, a common method in AI validation studies [9,21,25]. This was intentional and directly related to our goal of testing this AI not only as a mere replacement for the conventional Echo methodology in daily practice, but also as a “second pair of eyes”. Instead of simulating reality with a selection of representative Echo views, we allowed participating cardiologists to select their own Echo clips for MBS tracing, perform these online on the ultrasound machine and then send the complete Echo exam to the AI for fully automated view classification/selection and LVEF measurement. This gave us the opportunity to evaluate this AI-based application for clinical implementation. It is worth noting that the use of the AI-based application resulted in a longer time for LV function measurement compared to manual contouring with MBS. However, it is important to consider that the AI analysis includes fully automated view classification/selection, which can save time for the operator in selecting the optimal views and provide more time in individual patient care. Additionally, the increased time required for AI analysis may be justified by the potential for more consistent and accurate measurements. This is especially true in busy clinical settings, where variability in human operator measurements occur. Further research is needed to determine the optimal role of AI in clinical practice and to identify the most effective ways to integrate AI-based analysis into existing workflows.

### 4.4. Limitations

First, Echo datasets could not be selected for analysis for all patients. Thus, the accuracy of measurements determined by the AI-based application in subjects with very poor image quality could not be determined. Nevertheless, our prospective study resembles the real-world situation rather than a sterile experimental setup. Second, the intra-observer reliability on identical Echo datasets was not addressed. However, the test–retest agreement in the LVEF measurements yielded excellent and stable LVEF values for the AI. Third, due to the large cohort analyzed in our study, we were not able to compare the measurements to those from an independent imaging technique such as cardiac magnetic resonance in order to evaluate which method underestimates or overestimates the LVEF. However, recent studies suggest that human operators applying the MBS method tend to overestimate LVEF [9]. Finally, the AI-based application used in our study is a “closed tool,” and we do not have access to detailed information about the underlying CNN architecture. While this limits our ability to provide insights into the technical aspects of the application, it does not affect the validity of our findings regarding the clinical performance of the tool.

## 5. Conclusions

This novel seamless AI-based application automatically identified standard apical LV views and measured LVEF without user input in one workflow. It yielded similar results to the currently used MBS method and has the potential to improve LVEF quantification. Furthermore, it helps to reduce reader subjectivity. Despite the measurement bias, this AI method represents a viable option for use in a clinical setting.

## Figures and Tables

**Table 1:** Characteristics of the Studied Population (*N* = 889).

**Demographics**	
Age, years	71 [59; 80]
Male sex	542 (61)
**Anthropomorphic characteristics**	
Body mass index, kg/m²	27 [24; 29]
Heart rate, beats/min	70 [65; 76]
Systolic blood pressure, mm Hg	132 [102; 145]
Sinus rhythm	666 (75)
**Main Clinical Diagnosis**	
Valvular heart disease, severe	181 (20)
Aortic valve stenosis	73 (40)
Mitral valve regurgitation	57 (31)
Tricuspid valve regurgitation	41 (23)
Others	10 (6)
Coronary heart disease	236 (27)
Non-ischemic cardiomyopathy	88 (10)
Screening and clinical workup for	384 (43)
Suspected coronary artery disease	198 (52)
Suspected or known myocarditis	71 (18)
Others	115 (30)

Values are reported as *n* (%) or as median [interquartile range].

**Table 2:** Comparison of Measurement Results between AI and MBS (*N* = 889).

	AI	MBS	*p*-Value	Bias	*p*-Value	LOA [Lower; Upper]	ICC	R	*p*-Value
LVEF, %	48.7 [36.8; 56.2]	53.1 [42.2; 59.5]	<0.001	+4.5	<0.001	−8.6; 17.5	0.90	0.87	<0.001
LV EDV, mL	122 [87; 149]	98 [73; 130]	<0.001	−12	<0.001	−59; 34	0.90	0.89	<0.001
LV ESV, mL	56 [40; 86]	45 [31; 71]	<0.001	−11	<0.001	−41; 20	0.89	0.93	<0.001

Values are reported as the median [interquartile range]. AI = artificial intelligence; EF = ejection fraction; EDV = end-diastolic volume; ESV = end-systolic volume; ICC = intraclass correlation coefficient; LOA = limits of agreement; LV = left ventricular; MBS = modified biplane Simpson, R = Pearson’s correlation coefficient.

**Table 3:** Characteristics of the Main Clinical Diagnosis.

	Other *N* = 384	NICM *N* = 88	CHD *N* = 236	VHD *N* = 181	*p*-Value
Age, years	67 [54; 77]	64 [56; 72]	72 [64; 80]	78 [69; 83]	<0.001
Male sex	209 (54)	60 (68)	162 (69)	111 (61)	<0.001
Sinus rhythm	336 (88)	77 (88)	144 (61)	109 (60)	<0.001
Poor image quality	41 (11)	11 (10)	29 (12)	18 (10)	0.982
LVEF, %	59 [55; 63]	34 [26; 45]	44 [37; 48]	50 [36; 58]	<0.001
LV EDV, mL	79 [64; 99]	187 [150; 211]	99 [74; 123]	115 [86; 156]	<0.001
LV ESV, mL	33 [25; 43]	121 [96; 149]	53 [37; 70]	56 [39; 94]	<0.001

Values are reported as *n* (%) or the median [interquartile range]. LVEF, EDV and ESV were derived from this study using the MBS method. CHD = coronary heart disease; EDV = end-diastolic volume; LVEF = left ventricular ejection fraction; MBS = modified biplane Simpson; NICM = non-ischemic cardiomyopathy; VHD = valvular heart disease; ESV = end-systolic volume.

**Table 4:** Echocardiographic Characteristics of the Studied Population (*N* = 889).

**Categorization of Left Ventricular Ejection Fraction by MBS Method**	
>50%	534 (60)
40–49%	169 (19)
30–39%	111 (12)
<30%	75 (8)
**Echocardiographic image quality**	
Poor	99 (11)
Fair	258 (29)
Good	532 (60)

Values are reported as *n* (%). MBS = modified biplane Simpson.

**Table 5:** Reproducibility and Reliability (*N* = 40).

	AI		MBS		AI vs. MBS		AI vs. MBS	
	1st vs. 2nd Echo		1st vs. 2nd Echo		1st Echo		2nd Echo	
	ICC	COV	ICC	COV	ICC	Bias (unit)	ICC	Bias (unit)
LVEF	0.98	3.2%	0.89	5.9%	0.93	+4.5%	0.92	+4.7%
LV EDV	0.92	5.6%	0.89	7.1%	0.91	−13 mL	0.91	−15 mL
LV ESV	0.97	6.3%	0.94	11.5%	0.95	−9 mL	0.95	−13 mL

AI = artificial intelligence; COV = coefficient of variance; Echo = echocardiography; EF = ejection fraction; EDV = end-diastolic volume; ESV = end-systolic volume; ICC = intraclass correlation coefficient; LOA = limits of agreement; LVEF = left ventricular ejection fraction; EDV = end-diastolic volume; ESV = end-systolic volume; MBS = modified biplane Simpson.

## Data Availability

The data presented in this study are available on request from the corresponding author, as federal German law and ethics committee regulations prohibit public data sharing for the protection of patient security.

## Supplementary Materials

The following supporting information can be downloaded at: https://www.mdpi.com/article/10.3390/diagnostics13071298/s1.

## References

[1] Lang R.M., Badano L.P., Mor-Avi V., Afilalo J., Armstrong A., Ernande L., Flachskampf F.A., Foster E., Goldstein S.A., Kuznetsova T. (2015). Recommendations for cardiac chamber quantification by echocardiography in adults: An update from the American Society of Echocardiography and the European Association of Cardiovascular Imaging. J. Am. Soc. Echocardiogr..

[2] Aurich M., André F., Keller M., Greiner S., Hess A., Buss S.J., Katus H.A., Mereles D. (2014). Assessment of left ventricular volumes with echocardiography and cardiac magnetic resonance imaging: Real-life evaluation of standard versus new semiautomatic methods. J. Am. Soc. Echocardiogr..

[3] Hoffmann R., Barletta G., von Bardeleben S., Vanoverschelde J.L., Kasprzak J., Greis C., Becher H. (2014). Analysis of left ventricular volumes and function: A multicenter comparison of cardiac magnetic resonance imaging, cine ventriculography, and unenhanced and contrast-enhanced two-dimensional and three-dimensional echocardiography. J. Am. Soc. Echocardiogr..

[4] Knackstedt C., Bekkers S.C., Schummers G., Schreckenberg M., Muraru D., Badano L., Franke A., Bavishi C., Omar A.M.S., Sengupta P.P. (2015). Fully Automated Versus Standard Tracking of Left Ventricular Ejection Fraction and Longitudinal Strain the FAST-EFs Multicenter Study. J. Am. Coll. Cardiol..

[5] Thavendiranathan P., Grant A.D., Negishi T., Plana J.C., Popović Z.B., Marwick T.H. (2013). Reproducibility of Echocardiographic Techniques for Sequential Assessment of Left Ventricular Ejection Fraction and Volumes. J. Am. Coll. Cardiol..

[6] Asch F.M., Descamps T., Sarwar R., Karagodin I., Singulane C.C., Xie M., Tucay E.S., Rodrigues A.C.T., Vasquez-Ortiz Z.Y., Monaghan M.J. (2022). Human versus Artificial Intelligence–Based Echocardiographic Analysis as a Predictor of Outcomes: An Analysis from the World Alliance Societies of Echocardiography COVID Study. J. Am. Soc. Echocardiogr..

[7] Tromp J., Seekings P.J., Hung C.-L., Iversen M.B., Frost M.J., Ouwerkerk W., Jiang Z., Eisenhaber F., Goh R.S.M., Zhao H. (2022). Automated interpretation of systolic and diastolic function on the echocardiogram: A multicohort study. Lancet Digit. Health.

[8] Zhang J., Gajjala S., Agrawal P., Tison G.H., Hallock L.A., Beussink-Nelson L., Lassen M.H., Fan E., Aras M.A., Jordan C. (2018). Fully automated echocardiogram interpretation in clinical practice: Feasibility and diagnostic accuracy. Circulation.

[9] Samtani R., Bienstock S., Lai A.C., Liao S., Baber U., Croft L., Stern E., Beerkens F., Ting P., Goldman M.E. (2022). Assessment and validation of a novel fast fully automated artificial intelligence left ventricular ejection fraction quantification software. Echocardiography.

[10] Akkus Z., Aly Y., Attia I., Lopez-Jimenez F., Arruda-Olson A., Pellikka P., Pislaru S., Kane G., Friedman P., Oh J. (2021). Artificial intelligence (ai)-empowered echocardiography interpretation: A state-of-the-art review. J. Clin. Med..

[11] Bunting K.V., Steeds R.P., Slater L.T., Rogers J.K., Gkoutos G.V., Kotecha D. (2019). A Practical Guide to Assess the Reproducibility of Echocardiographic Measurements. J. Am. Soc. Echocardiogr..

[12] Quer G., Arnaout R., Henne M., Arnaout R. (2021). Machine Learning and the Future of Cardiovascular Care: JACC State-of-the-Art Review. J. Am. Coll. Cardiol..

[13] Litjens G., Ciompi F., Wolterink J.M., de Vos B.D., Leiner T., Teuwen J., Išgum I. (2019). State-of-the-Art Deep Learning in Cardiovascular Image Analysis. JACC Cardiovasc. Imaging.

[14] Asch F.M., Poilvert N., Abraham T., Jankowski M., Cleve J., Adams M., Romano N., Hong H., Mor-Avi V., Martin R.P. (2019). Automated Echocardiographic Quantification of Left Ventricular Ejection Fraction Without Volume Measurements Using a Machine Learning Algorithm Mimicking a Human Expert. Circ. Cardiovasc. Imaging.

[15] Kusunose K., Haga A., Yamaguchi N., Abe T., Fukuda D., Yamada H., Harada M., Sata M. (2020). Deep Learning for Assessment of Left Ventricular Ejection Fraction from Echocardiographic Images. J. Am. Soc. Echocardiogr..

[16] Bhuva A.N., Bai W., Lau C., Davies R.H., Ye Y., Bulluck H., McAlindon E., Culotta V., Swoboda P.P., Captur G. (2019). A Multicenter, Scan-Rescan, Human and Machine Learning CMR Study to Test Generalizability and Precision in Imaging Biomarker Analysis. Circ. Cardiovasc. Imaging.

[17] Yuan N., Jain I., Rattehalli N., He B., Pollick C., Liang D., Heidenreich P., Zou J., Cheng S., Ouyang D. (2021). Systematic Quantification of Sources of Variation in Ejection Fraction Calculation Using Deep Learning. JACC Cardiovasc. Imaging.

[18] Ghorbani A., Ouyang D., Abid A., He B., Chen J.H., Harrington R.A., Liang D.H., Ashley E.A., Zou J.Y. (2020). Deep learning interpretation of echocardiograms. Npj Digit. Med..

[19] Playford D., Bordin E., Mohamad R., Stewart S., Strange G. (2020). Enhanced Diagnosis of Severe Aortic Stenosis Using Artificial Intelligence: A Proof-of-Concept Study of 530,871 Echocardiograms. JACC Cardiovasc. Imaging.

[20] Pellikka P.A., Strom J.B., Pajares-Hurtado G.M., Keane M.G., Khazan B., Qamruddin S., Tutor A., Gul F., Peterson E., Thamman R. (2022). Automated analysis of limited echocardiograms: Feasibility and relationship to outcomes in COVID-19. Front. Cardiovasc. Med..

[21] Salte I.M., Østvik A., Smistad E., Melichova D., Nguyen T.M., Karlsen S., Brunvand H., Haugaa K.H., Edvardsen T., Lovstakken L. (2021). Artificial Intelligence for Automatic Measurement of Left Ventricular Strain in Echocardiography. JACC Cardiovasc. Imaging.

[22] Otani K., Nakazono A., Salgo I.S., Lang R.M., Takeuchi M. (2016). Three-Dimensional Echocardiographic Assessment of Left Heart Chamber Size and Function with Fully Automated Quantification Software in Patients with Atrial Fibrillation. J. Am. Soc. Echocardiogr..

[23] Zhang J., Chatham J.C., Young M.E. (2020). Circadian Regulation of Cardiac Physiology: Rhythms That Keep the Heart Beating. Annu. Rev. Physiol..

[24] Houard L., Militaru S., Tanaka K., Pasquet A., Vancraeynest D., Vanoverschelde J.L., Pouleur A.C., Gerber B.L. (2021). Test-retest reliability of left and right ventricular systolic function by new and conventional echocardiographic and cardiac magnetic resonance parameters. Eur. Heart J. Cardiovasc. Imaging.

[25] Cannesson M., Tanabe M., Suffoletto M.S., McNamara D.M., Madan S., Lacomis J.M., Gorcsan J. (2007). A Novel Two-Dimensional Echocardiographic Image Analysis System Using Artificial Intelligence-Learned Pattern Recognition for Rapid Automated Ejection Fraction. J. Am. Coll. Cardiol..

