# Supplementary material for: Single-Site Experience with an Automated Artificial Intelligence Application for Left Ventricular Ejection Fraction Measurement in Echocardiography
Source: Diagnostics (Basel). 2023 Mar 30;13(7):1298. doi: 10.3390/diagnostics13071298 (PMC10093353; doi:10.3390/diagnostics13071298)
Supplement: Supplementary file 1 [file diagnostics-13-01298-s001.zip › diagnostics-2227452-supplementary.pdf]

Supplementary Materials

Single-site experience with an automated artificial intelligence application for left ventricular ejection fraction measurement in echocardiography

Krunoslav Michael Sveric *, Roxana Botan, Zouhir Dindane, Anna Winkler, Thomas Nowack, Christoph Heitmann, Leonhard Schleußner and Axel Linke

Department of Internal Medicine and Cardiology, Herzzentrum Dresden, Technische Universität Dresden, Fetscherstr. 76, 01307 Dresden, Germany

* Correspondence: kruno.sveric@caroconnect.de

Supplemental Figures

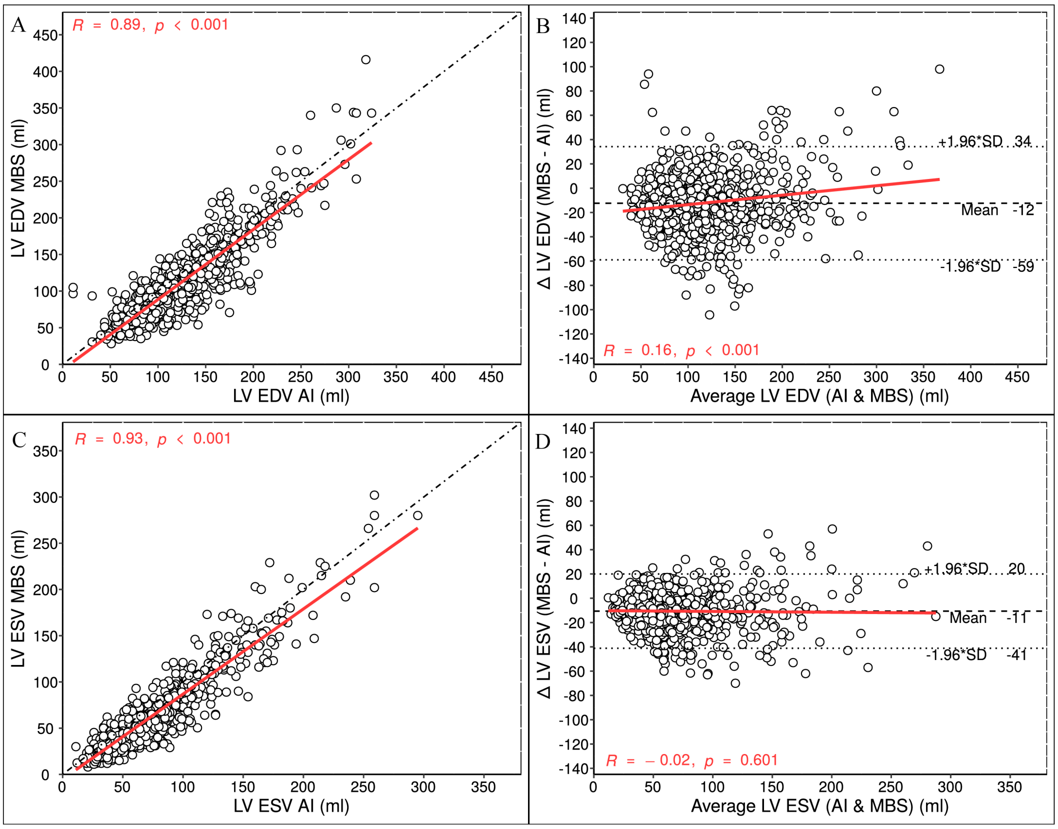

Supplemental Figure S1:
Title: Comparison of LV Volumes for the AI-based application and MBS method
Caption: Correlations (A and C) and Bland-Altman analyses (B and D) of the LV EDV (upper row) and LV ESV (lower row) of AI-based application vs. MBS technique.
In correlations dashed-dotted lines represent the line of identity. In Bland-Altman analyses dashed horizontal lines represent the mean bias and dotted horizontal lines represent the upper and lower limits of agreement as ±1.96 * standard deviation (SD). R denotes Pearson's correlation coefficient with the

corresponding significance value p. AI = artificial intelligence; EDV = end-diastolic volume; ESV = end-systolic volume; LV = left ventricular; MBS = modified biplane Simpson.

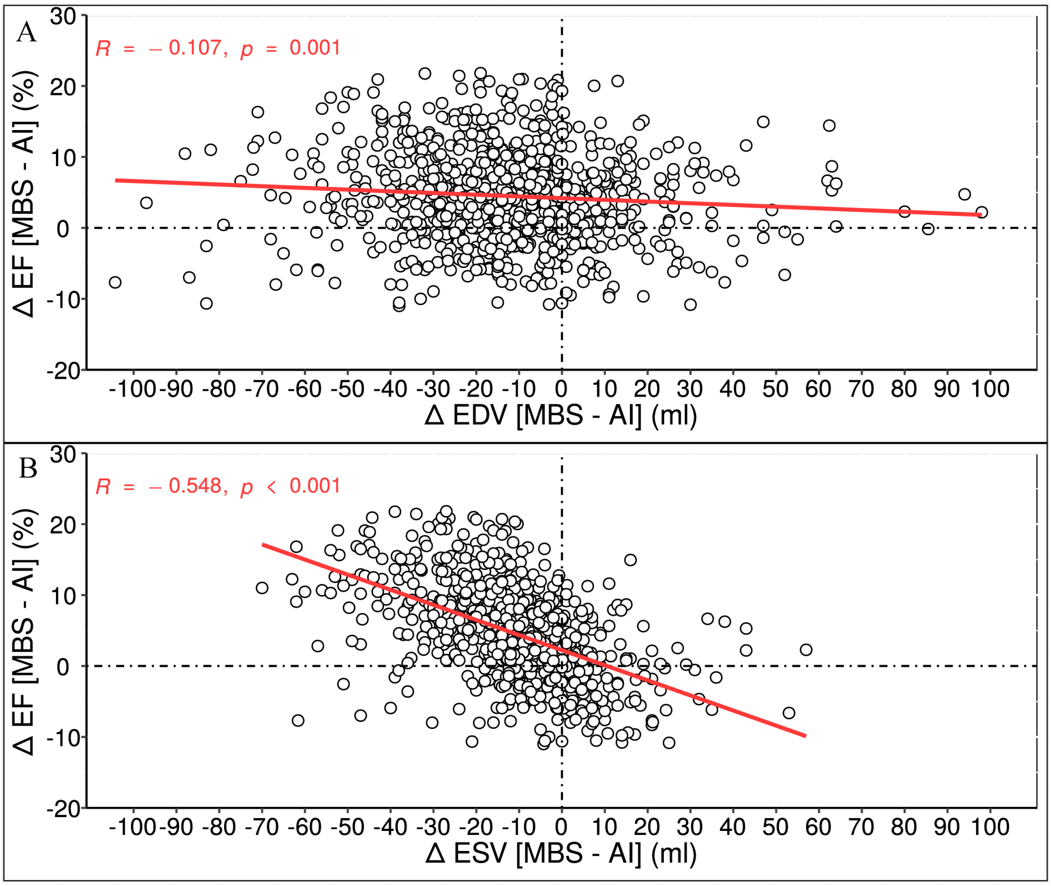

Supplemental Figure S2:

Title: LV EF bias in dependence on measurement differences of LV volumes between AI-based application and MBS method

Caption: Correlations for differences in EDV (A) and ESV (B). R denotes Pearson's correlation coefficient with the corresponding significance value p.

AI = artificial intelligence; EF = ejection fraction; EDV = end-diastolic volume; ESV = end-systolic volume; LV = left ventricular; MBS = modified biplane Simpson.
